# Influencing factors of reproductive concerns in reproductive-aged male patients with cancer: a systematic review

**DOI:** 10.3389/fpubh.2026.1803648

**Published:** 2026-05-01

**Authors:** Changmin Niu, Xueyu Lu, Fan Yang, Shikun Ma, Yuxin Gao, Nuo Shen, Wenxuan Zhu, Yidi Kong, Lu Chen, Junli Yan

**Affiliations:** 1 Department of Gynaecology and Obstetrics, Affilited Hospital of Yangzhou University, Yangzhou, Jiangsu, China; 2 School of Nursing, Faculty of Medicine, Yangzhou University, Yangzhou, China; 3 School of Basic Medical Sciences and School of Public Health, Faculty of Medicine, Yangzhou University, Yangzhou, China; 4 Yangzhou Hospital of Traditional Chinese Medicine, Yangzhou, Jiangsu, China; 5 Department of Anesthesiology, Affilited Hospital of Yangzhou University, Yangzhou, Jiangsu, China; 6 Department of Nursing, Affilited Hospital of Yangzhou University, Yangzhou, Jiangsu, China

**Keywords:** cancer, influencing factors, male patients with cancer, reproductive age, reproductive concerns

## Abstract

**Objectives:**

Advancements in medical technology have significantly improved cancer treatment outcomes, enabling an increasing number of patients to survive long-term post-treatment. However, chemotherapy, radiotherapy, and surgery frequently cause varying degrees of damage to the male reproductive system, which affects fertility and leads to varying degrees of reproductive concerns in patients. This study aimed to review the current state and factors influencing reproductive concerns in reproductive-aged male patients with cancer, explore potential support requirements for this population, and lay the theoretical foundation for formulating individualized intervention measures.

**Methods:**

A systematic literature search of databases (PubMed, MEDLINE, Cochrane, Web of Science, Embase, EBSCO, CNKI, Wan-fang data, and VIP) was conducted from their inception to December 2024.

**Results:**

The final analysis included 15 studies involving 2,741 patients. The summary indicates that reproductive concerns among reproductive-aged male patients with cancer are moderately elevated, leading to negative emotions, including anxiety and depression. The reproductive concerns of these patients are predominantly influenced by six factors: sociodemographic factors, cancer and related treatments, reproduction and fertility, the quality of social relationships, fertility preservation, and psychological factors.

**Conclusion:**

Reproductive-aged male patients with cancer commonly experience moderately high levels of reproductive concerns. Prolonged exposure to moderate-to-high levels of reproductive concerns is associated with the onset of mental health issues and the potential adverse impact on cancer treatment. Therefore, clinical healthcare providers must address these specific psychological challenges, intervene in existing reproductive concerns, and assist patients in better accepting and adapting to cancer and related treatments.

**Systematic review registration:**

https://www.crd.york.ac.uk/prospero/display_record.php?ID=CRD42024565381, Identifier, CRD42024565381.

## Highlights

- Evaluates the current state and influencing factors of reproductive concerns in reproductive-aged male patients with cancer by systematic review.
- Identifies potential support needs for this population and provide a theoretical foundation for developing effective interventions.
- Makes up the lack of systematic reviews of descriptive studies on male cancer patients of reproductive age.

## 1 Introduction

The global rise in cancer risk is resulting in increased incidence and mortality rates among young patients (1). GLOBOCAN’s(Global Cancer Observatory)recently released estimates have indicated that approximately 19.3 million new cancer cases and nearly 10 million deaths occurred worldwide in 2020, with an expected increase to 28.4 million cases by 2040, representing a 47% growth rate (2). Alongside this staggering growth rate, cancer is increasingly impacting younger individuals, including those who are unmarried or childless (3). Owing to the increasing trend of late marriage and childbearing, particularly in developed countries, some patients diagnosed with cancer have not yet completed their fertility plans (4). However, cancer treatment can lead to gonadal damage, which is widely recognized. These effects may be directly caused by surgery, radiotherapy, cytotoxic chemotherapy, or mediated by hormonal changes (5). Moreover, studies have indicated that with the evolution of human genes, cancer incidence increases, and male fertility is affected (6). Adverse reactions to cancer treatment further affect fertility, making fertility a significant concern for male patients with cancer during treatment (6).

The World Health Organization defines reproductive age as starting at 15 and ending at 49 years (7). Reproductive-aged male patients with cancer are still young and aspire to continue living. As cancer is diagnosed at a younger age and survival periods are extended, many patients with cancer who have not yet completed their fertility plans may face fertility decisions (8). The concept of reproductive concerns was first proposed by Wenzel in 2005, primarily targeting young female patients with cancer and gynecological tumors and lymphomas, having concerns focused on symptoms and emotions (9). Subsequently, Gorman discovered that patients’ apprehensions extended beyond their condition and involved their children’s cancer risk and health concerns, leading to an expansion of the dimensions of reproductive concerns to include worries about children and partners (10). This concept was then extended to male patients with cancer, including those with lymphoma, lung cancer, rectal cancer, and testicular cancer. Research has confirmed that young patients with cancer are prone to reproductive concerns, which can severely impact their psychosocial health and treatment decisions (11). Moderately high levels of reproductive concerns are closely related to negative emotions, including depression and sadness, harming patients’ self-esteem and leading to psychological problems. Additionally, it can undermine relationship satisfaction with partners, ultimately reducing emotional relationships and quality of life (12). Furthermore, reproductive concerns can decrease patients’ adherence to cancer treatment, hindering rational decision-making regarding personal treatment plans, potentially leading to missed optimal treatment opportunities, and posing significant threats to patients’ physical health (11, 12).

Scholars from multiple countries have recently conducted extensive research on reproductive concerns among patients with cancer, including cross-sectional and qualitative studies, to better understand their status, influencing factors, and patients’ true inner experiences (13). However, research on reproductive concerns is predominantly focused on female patients with cancer (14), with relatively few studies on male patients. Although cancer has a significant impact on the fertility of young women, the fertility needs of other cancer groups should not be overlooked. Data reveals that globally, more than 50% of young male patients with cancer may experience impaired fertility after treatment, with some facing a lifetime risk of infertility (15). Currently, conclusions regarding reproductive concerns among reproductive-aged male patients with cancer across various cancer types are inconsistent (11). The assessment tools for this population are still not well-developed, and most of the assessment tools target female cancer patients (16). Besides, descriptive studies on reproductive concerns among reproductive-aged male patients with cancer lack systematic evaluation, hindering clinical healthcare providers’ understanding of the current status and influencing factors, thus preventing effective interventions (17).

Therefore, this systematic review aims to: (1) reveal the current status of reproductive concerns among reproductive-aged male patients with cancer; (2) Determine the relevant influencing factors; (3) To supplement the lack of attention to fertility among male cancer patients, with the aim of identifying high-risk patients as early as possible and exploring the potential support needs of this group, providing a theoretical basis for formulating complete and individualized intervention measures, thereby reducing the level of fertility concerns among male cancer patients of childbearing age.

## 2 Materials and methods

### 2.1 Design

This systematic review complies with the Preferred Reporting Items for Systematic Reviews and Meta-analyses (PRISMA) statement.

The review protocol and records are available online through the Prospective Register of Systematic Reviews (PROSPERO 2024 CRD42024565381) and is available online through the International Prospective Register of Systematic Reviews.^1^ The detailed registration report can be found in Supplementary files.

### 2.2 Search strategy

A comprehensive search was conducted in PubMed, the Cochrane Library, Web of Science, Embase, Medline, CNKI, Wanfang Database, and VIP Database. To ensure the comprehensiveness and accuracy of the search, the core concepts, subject terms, and free words in all database searches are kept consistent. Since the same subject terms were used in the database search, the search strategy is detailedly listed as an example using the representative database PubMed.

The search terms in PubMed were as follows: (“male cancer survivors” OR “men with cancer” OR “male adolescents” OR “man cancer”) AND (“reproductive* concerns” OR “reproductive anxiety” OR “reproductive worries” OR “fertility worries” OR “fertility-related distress” OR “Infertility-related distress”) AND (“risk factors” OR “related factors” OR “relevant factors” OR “influencing factors”) OR (“influence factors” OR “predictors” OR “predictive factors”). We also identified eligible articles from the references. The search period was from the inception of the databases to December 2024. Both subject terms and free terms were used. To minimize the risk of missing relevant studies, references of the included literature and related systematic reviews were tracked (see Table 1 for details).

**Table 1:** Search strategy of the representative database PubMed.

Number	Search terms
*#*1	((“Male”[MeSH Terms] OR “men”[Title/Abstract] OR “male adolescents”[Title/Abstract] OR “males”[Title/Abstract]) AND (“Cancer Survivors”[MeSH Terms] OR “Neoplasms”[MeSH Terms] OR “cancer”[Title/Abstract] OR “neoplasm*”[Title/Abstract]) AND (survivor*[Title/Abstract] OR “cancer survivor*”[Title/Abstract]))
*#*2	((“Reproduction”[MeSH Terms] OR “Reproductive Health”[MeSH Terms] OR “Fertility”[MeSH Terms] OR “reproduction” [Title/Abstract] OR “fertility” [Title/Abstract] OR concern*[Title/Abstract] OR “worry” [Title/Abstract] OR “worries” [Title/Abstract] OR “anxiety” [Title/Abstract] OR “distress” [Title/Abstract] OR “reproductive concerns”[Title/Abstract] OR “fertility-related distress”[Title/Abstract]))
*#*3	((“Risk Factors”[MeSH Terms] OR “risk factor*”[Title/Abstract] OR predictor*[Title/Abstract] OR “associated factor*”[Title/Abstract] OR “influencing factor*”[Title/Abstract] OR “influence factor*”[Title/Abstract] OR “related factor*”[Title/Abstract] OR “relevant factor*”[Title/Abstract] OR “predictive factor*”[Title/Abstract]))
*#*4	#1 AND #2 AND #3

### 2.3 Inclusion and exclusion criteria

The inclusion criteria were as follows: (a) Studies on male cancer patients confirmed by pathological examination; (b) Patients aged between 15 and 49 years; (c) Observational studies (case–control studies, cohort studies, descriptive studies, etc.); (d) Studies where the main outcome measure was fertility concerns. (e) Chinese and English studies.

Exclusion criteria included: (a) Studies with insufficient data; (b) Non-English or non-Chinese studies; (c) Conference papers, duplicate publications, incomplete information, or studies without full-text access.

### 2.4 Literature screening and data extraction

First, import all the retrieved manuscripts into the NoteEx-press software and automatically delete and restore the manuscripts. The two researchers of the review screened the studies independently and extracted data based on inclusion and exclusion criteria. These differences were resolved through discussion or consultation with independent third-party scientists. Excerpts include: basic information about the study (such as author, year of publication, study location, etc.); Key characteristics of the subject (such as age, education, marital status, etc.); Research methods and evaluation tools; The status of reproductive problems and their influencing factors (see Table 2 for details).

**Table 2:** Characteristics of included studies (*n* = 15).

Author and Year	Country	Type of study	Type of patient	Mean age	Number of patient	Research tool	Influencing factors
Liao C, 024 (18)	China	Cross-sectional study	Male cancer patient	30	389	RCAC-M	②③④⑤⑦⑨⑭⑮⑳
LihuaWu, 2024 (19)	China	Cross-sectional study	Male cancer patient	29.6	369	RCAC-M	①⑥⑩⑪⑫⑯
Yan Hu, 2024 (20)	China	Cross-sectional study	Middle-aged urological cancer patients	38	99	RCAC-M	②⑥⑤⑭⑳
Yangshen Feng, 2024 (21)	China	Cross-sectional study	Male cancer patient	33.76±7.5	236	RCAC-M	①②③④⑨⑮
Chiu Yi Tan, 2024 (22)	China	Cross-sectional study	Male cancer patient	19	17	RCAC	①②⑦⑲
Richard Nyeko, 2024 (23)	Africa	Cross-sectional study	Male cancer patient	20.1	59	MRCS	①⑦⑨⑱
Brigitte G, 2023 (24)	Australia	Case control study	Male cancer patient	24.5	58	RCAC	⑥⑩⑪⑫
Kenny A, 2023 (25)	Sweden	Cohort study	Male cancer patient	32.1±5.5	316	RCAC	③④⑦⑩⑪⑬⑱⑲㉑
Jie Liu, 2023 (26)	China	Cross-sectional study	Male cancer patient	36.1±3.0	447	RCAC	⑨⑪⑯
Julia H, 2021 (27)	America	Cross-sectional study	Male cancer patient	28.1	170	RCAC	⑥⑪⑯
Jessica R, 2020 (28)	England	Cross-sectional study	Male cancer patient	18-35	170	RCAC-M	⑨⑪⑯
Ljungman L, 2019 (29)	Swede	Cross-sectional study	Male cancer patient	26.2	111	RCAC	⑩⑪⑬⑯㉓
Jane M, 2019 (30)	America	Cross-sectional study	Male cancer patient	25.5	185	FPI	①④⑧⑯⑰㉓
Tatsuro F, 2018 (31)	Japan	Cross-sectional study	Male cancer patient	21.3	100	Self-designed questionnaire	⑥⑧㉒
Jane M, 2018 (32)	America	Cross-sectional study	Male cancer patient	42.53	185	FPI	⑩⑪⑮⑰

### 2.5 Quality assessment

To ensure the credibility and methodological rigor of the included studies, we employed the Strengthening the Reporting of Observational Studies in Epidemiology (STROBE) (55) checklist to assess the quality of the 15 eligible articles. The STROBE statement is an internationally recognized guideline for evaluating observational studies, including cross-sectional, case–control, and cohort designs. It comprises 22 criteria covering key domains such as study design clarity, population definition, variable measurement, statistical methodology, bias control, and result interpretation. Two independent reviewers, both trained in evidence-based methodology, performed the assessments separately. Disagreements were resolved through discussion or arbitration by a third senior reviewer. This guide contains 22 points for assessing the quality of cross-sectional and case–control studies. Each gets 1 point if the guidelines are met, and 0 points if the description is wrong. The possible total is 22 points. ≥17 studies were of high quality, 11 to 16 were of medium quality, and ≤10 is were low quality. Thirteen articles were assessed as high quality and two as moderate quality (see Table 3 for details).

**Table 3:** Quality evaluation results of included studies.

Author and Year	**Title and abstract**	**Introduction**	**Methods**	**Results**	**Discussion**	**Other information**	**Score**	**Quality of evidence**
	**(1)**	**(2)**	**(9)**	**(5)**	(4)	**(1)**		
Liao C, 2024 (18)	1	2	7	4	3	0	17	High
LihuaWu, 2024 (19)	1	2	7	4	3	1	18	High
Yan Hu, 2024 (20)	1	2	7	4	3	0	17	High
Yangshen Feng, 2024 (21)	1	2	8	4	4	0	19	High
Chiu Yi Tan, 2024 (22)	1	2	7	4	3	1	18	High
Richard Nyeko, 2024 (23)	1	2	6	3	3	1	16	Moderate
Brigitte G, 2023 (24)	1	2	8	4	3	1	19	High
Kenny A, 2023 (25)	1	2	8	4	3	1	19	High
Jie Liu, 2023 (26)	1	2	7	4	4	0	18	High
Julia H, 2021 (27)	1	2	8	4	3	1	19	High
Jessica R, 2020 (28)	1	2	7	3	3	1	17	High
Ljungman L, 2019 (29)	1	2	7	3	3	1	17	High
Jane M, 2019 (30)	1	2	7	4	3	1	18	High
Tatsuro F, 2018 (31)	1	2	6	3	3	1	16	Moderate
Jane M, 2018 (32)	1	2	7	4	3	1	18	High

## 3 Results

### 3.1 Literature search results

**Figure 1:**
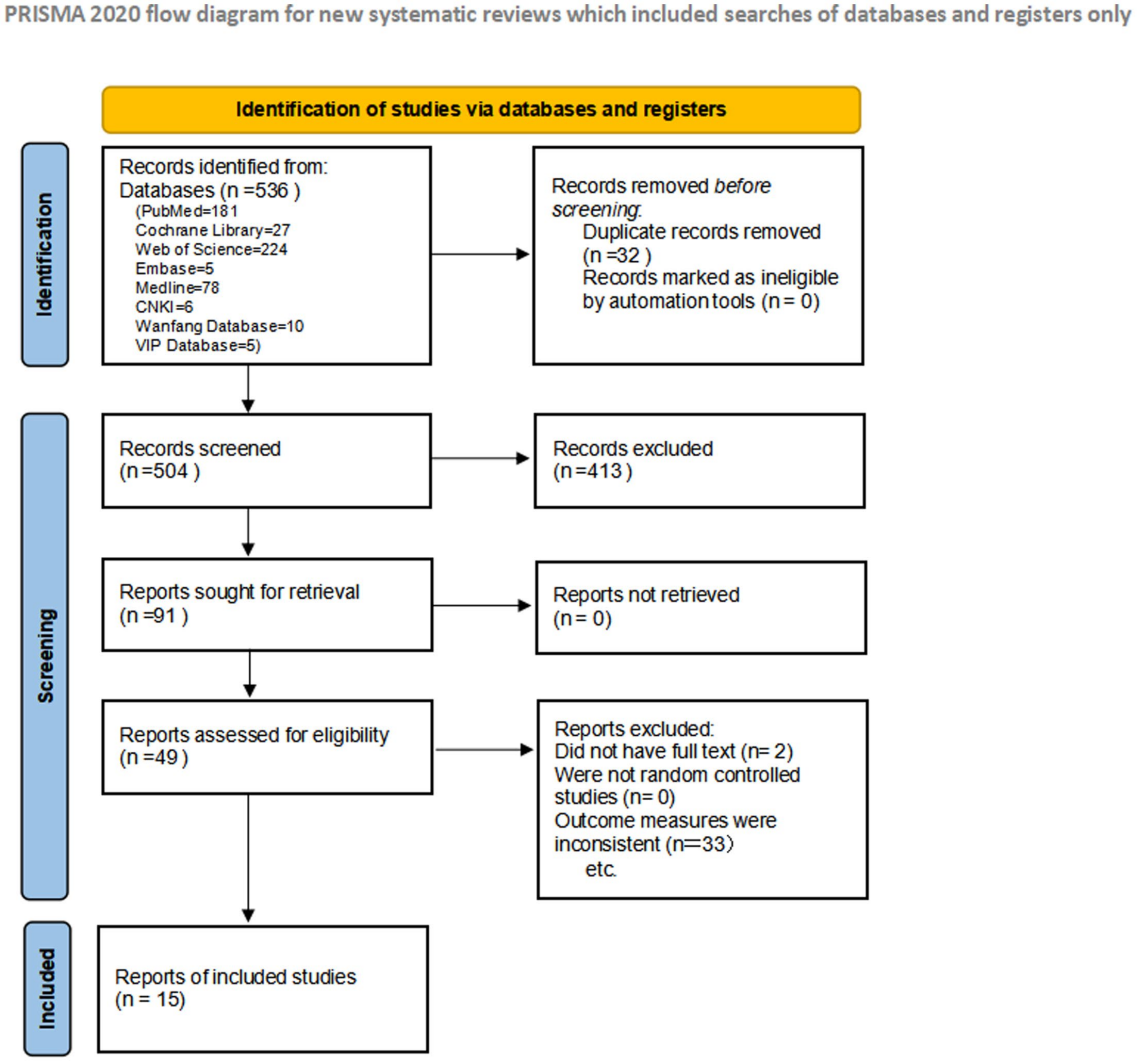
Flow diagram of the review process.

The initial search yielded 536 articles. As shown in the PRISMA flow diagram (Figure 1), the search of electronic databases generated a total of 536 records. This total comprised 181 records from PubMed, 27 from the Cochrane Library, 224 from Web of Science, 5 from Embase, 78 from Medline, 6 from CNKI, 10 from the Wanfang Database, and 5 from the VIP Database. After deduplication using Endnote software, 504 articles remained. Title and abstract screening excluded 413 articles as irrelevant. Full-text screening of the remaining 91 articles excluded 78 for various reasons (such as reviews, qualitative studies, etc.). Ultimately, 15 studies were included in the systematic review, comprising 2,741 patients (11, 18–31). These included one cohort study (25), one case–control study (24), and thirteen studies (11, 18–23, 26–31). Measurement tools for fertility concerns included the Reproductive Concerns After Cancer Scale (RCAC), the Fertility Issues and Outcomes Scale (FIS), and the Reproductive Concerns Scale (RCS). Figure 1 summarizes the study selection process.

### 3.2 Characteristics and quality assessment of included studies

The fundamental features of the included literature are summarized in Table 2. The sample size was 2,741, with samples from the United Kingdom, United States, Australia, Sweden, Japan, China, and African countries. One study used the MRCS (10-item Modified Reproductive Concerns Scale), two used the FPI, and one used a self-designed fertility questionnaire. Furthermore, five studies used Reproductive Concerns After Cancer (RCAC-M) as their primary measurement tool, and seven used RCAC as their primary measurement tool. The present situation and the factors influencing fertility anxiety are the subjects addressed.

## 4 Current status of reproductive concerns in reproductive-aged male patients with cancer

### 4.1 Prevalence of reproductive concerns in reproductive-aged male patients with cancer

A survey in the United States indicated that over 60% of reproductive-aged male patients with cancer exhibited high scores for reproductive concerns (27), with more than half attributing their worries to multiple factors. Similarly, a latent profile analysis study in China demonstrated that fertility concerns among reproductive-aged male patients with cancer were moderately elevated (21), consistent with the findings by American researcher Drizin in his study on male cancer survivors (27). A Swedish cohort study reported that 27% of males exhibited high fertility concerns on at least one dimension of the RCAC scale 1.5 years post-cancer diagnosis (25). Additional studies suggest that these patients are apprehensive about communication with their partners, including but not limited to future fertility plans (27, 29). Furthermore, concerns regarding the health of future children potentially affected by genetic factors associated with the disease and personal health issues constitute major sources of reproductive concerns for these individuals (24, 25, 27).

### 4.2 Impact of reproductive concerns on the physical and mental health of reproductive-aged male patients with cancer

Reproductive concerns can lead to long-term psychological stress, persisting from cancer diagnosis and potentially having more lasting effects than cancer and its treatments (10). Studies indicate that moderate to high levels of reproductive concerns are associated with negative emotions, including sadness, resentment, and depression, lowering self-esteem, reducing partner relationship satisfaction, and ultimately decreasing the overall quality of life (11). In addition, Takeuchi discovered that reproductive concerns may reduce adherence to cancer treatments, hindering patients from making rational treatment decisions and potentially missing optimal treatment opportunities, thereby posing significant health threats (12). Therefore, healthcare providers should address the specific psychological issues of these patients, assisting them in better accepting and adapting to cancer and its treatment.

## 5 Influencing factors of reproductive concerns

### 5.1 Sociodemographic factors

The sociodemographic factors influencing reproductive concerns in reproductive-aged male patients with cancer included age, education level, marital status, and whether they have children.

Five studies reported that younger reproductive-aged male patients with cancer were more likely to experience higher levels of reproductive concerns (11, 19, 21–23). Researchers have indicated that males aged 21–31 years have the highest levels of fertility concerns post-cancer (21). Four additional studies demonstrated that individuals with higher education levels were more susceptible to high reproductive concerns (18, 19, 21, 22). Highly educated male patients with cancer often exhibited a stronger sense of family responsibility and paternal role identification, making them more attentive and sensitive to fertility issues (20). In addition, childlessness was a significant factor influencing reproductive concerns, regardless of the patient’s age or tumor type (11, 18, 21, 25). Additionally, marriage and parenthood served as protective factors against reproductive concerns, whereas unmarried and childless individuals were more likely to experience moderate to high levels of reproductive concerns (18, 21, 25).

### 5.2 Cancer and related treatments

Factors affecting reproductive concerns in reproductive-aged male patients with cancer include disease stage, treatment methods, cancer type, and time of diagnosis. Higher disease stages are associated with greater reproductive concerns due to the worse condition and uncertain prognosis, which may lead to concerns about long-term survival and stronger reproductive concerns (18, 20). Higher disease stages often require more aggressive treatments, including surgery, radiation therapy, and chemotherapy, which can significantly impact fertility (32). Research by Ljungman indicates higher reproductive concerns in patients undergoing chemotherapy and endocrine therapy due to the side effects (29). Besides, different cancer types contribute to varying levels of reproductive concern (22, 23, 25). Common cancers in reproductive-age men include leukemia (19%), gastrointestinal cancer (14%), and testicular cancer (12%) (27). Men undergoing brain tumor treatment experience fertility concerns more than four times higher than those treated for testicular cancer, likely because of the malignancy and complexity of brain tumor treatments (25).

### 5.3 Reproductive factors

The factors related to reproduction and fertility that influence reproductive-aged male patients with cancer include fertility desires, reproductive potential, concerns about the health of future children, the genetic risk of cancer, and personal health concerns. There is a positive correlation between fertility desires and fertility concerns; the stronger the fertility desire, the higher the level of reproductive concerns (18, 21). Research has revealed that up to 80% of reproductive-aged male patients with cancer express strong fertility desires after cancer diagnosis, particularly those who have not yet had children (33). Unfulfilled fertility desires are one of the most common reasons for reproductive concerns (11). Multiple survey studies indicate that the most common fertility-related worries among men include concerns about the health of existing or future children, the hereditary risk of cancer, and their reproductive potential (11, 19, 24, 25, 27, 29, 31). In a multivariate analysis, compared to women, male cancer survivors were more likely to worry about whether their medical history would affect the health of their existing or future children and expressed more concern about passing on cancer risks to potential offspring (19, 24). The desire to have children in the future is positively correlated with reproductive concerns, with those wanting children more likely to worry about their reproductive potential. Research indicates that middle-aged patients aged 31–41 years are more concerned about their personal health being affected by reproduction, fearing that their illness may prevent them from caring for their children, thereby exacerbating reproductive concerns (25, 27).

### 5.4 Quality of social relationships

The factors related to the quality of social relationships that influence reproductive concerns in reproductive-aged male patients with cancer include the level of life quality, peer support, partner communication, and personal life satisfaction. Research indicates that the higher the quality of life among middle-aged patients with urological cancer, the lower their level of reproductive concerns (20), consistent with findings by British scholar Park (34). Regression analysis indicates that lower levels of peer support are associated with higher reproductive concerns. Male patients with cancer often experience feelings of shame and inferiority, reluctant to seek help and support, believing that their spouses, parents, and friends cannot understand or do not need to understand their emotions, resulting in a lack of support and comfort in their family and social circles (18, 21, 31) Other studies reveal that sexual dysfunction and fertility-related distress are prevalent among reproductive-aged male patients with cancer post-treatment. Approximately 50% of patients report sexual dysfunction a year after cancer diagnosis, leading to decreased sexual satisfaction (11). Three studies indicated that reproductive-aged male patients with cancer reported high levels of anxiety in the “partner communication” dimension (26, 28). Gorman used the RCAC-M scale to assess the reproductive concerns of 170 young male patients with cancer, finding that the partner communication dimension scored the highest among all dimensions (28). This suggests that effective communication with partners regarding fertility issues and maintaining good partner relationships remain the most challenging and concerning issues for reproductive-aged male patients with cancer. A systematic review in China also pointed out that positive support from partners is one of the essential resources for reproductive-aged patients to effectively cope with infertility-related issues (35).

### 5.5 Fertility counseling (FC) and preservation

Fertility counseling involves assessing the potential fertility risks posed by cancer and related treatments for reproductive-aged patients, recommending appropriate fertility preservation methods based on the patient’s situation, and assisting patients in making reproductive treatment decisions (36). Research indicates that over half of reproductive-aged male patients with cancer were not adequately informed about the potential fertility risks of cancer and its treatments before initiating treatment, significantly hindering the orderly conduct of subsequent fertility preservation efforts (37). A survey by Zhang et al. (38) revealed that 99.2% of reproductive-aged male patients with cancer expressed a desire to receive fertility health information from healthcare providers. Other studies indicate that male cancer survivors who received FC were more likely to experience significant fertility issues compared to those who did not receive FC, suggesting that the current quality of FC is insufficient to alleviate their reproductive concerns (27). Research reveals that fertility preservation can buffer the growing reproductive concerns among patients with cancer (26). Methods of male fertility preservation include ultra-low temperature freezing of peripheral sperm, with peripheral sperm cryopreservation being a well-established method for this purpose (39). Wang’s study indicated that successful fertility preservation can effectively alleviate the reproductive concerns of reproductive-aged male patients with cancer, reducing their regret over fertility and improving their quality of life and confidence in combating the illness (40). However, most patients and healthcare providers possess a relatively limited understanding of fertility preservation. Katz’s investigation of 200 young male cancer survivors revealed that only 51% underwent sperm cryopreservation after cancer treatment (41).

### 5.6 Psychological factors

The psychological factors influencing reproductive concerns in reproductive-aged male patients with cancer include the level of disease perception, self-efficacy and self-disclosure, fear of disease progression, and disease acceptance. Research has demonstrated that increased disease perception levels are correlated with higher (20) reproductive concerns, corroborated by research from Gao et al. (42). In psychological factors, high anxiety and low self-efficacy are associated with high reproductive concerns, and increased self-disclosure has been found to be associated with low fertility concerns, consistent with previous findings in young women with cancer (19). Fear of disease recurrence refers to the negative emotions of worry and fear about the recurrence or worsening of the disease (43). The fertility-related decisions of reproductive-aged male patients with cancer are readily influenced by factors including fear of disease recurrence, especially among patients with high recurrence rates of hematologic tumors (20). Studies indicate that patients often avoid implementing fertility plans post-cancer treatment due to fear that reproduction may lead to disease recurrence. These unfulfilled fertility desires lead to or exacerbate reproductive concerns (44). Moreover, disease acceptance is considered a critical factor in managing chronic illnesses (including cancer), with higher acceptance of the disease correlating with lower levels of reproductive concerns (25). Research on female reproductive concerns has already demonstrated that disease acceptance is a protective factor against fertility concerns (11, 45).

## 6 Discussion

### 6.1 Analysis current status of reproductive concerns in reproductive-aged male patients with cancer

This systematic review reported that reproductive concerns are generally at a moderately high level among reproductive-aged male patients with cancer. Research has confirmed that males exhibit higher fertility motivations than females (45), and individuals diagnosed with malignant tumors are likely to experience elevated reproductive concerns. Persistent reproductive concerns can lead to negative emotions, which may adversely affect patients’ health. Reproductive concerns among reproductive-aged male patients with cancer span physiological, psychological, and social dimensions, including concerns about impaired fertility and health at the physiological level, worries about reproductive potential and children’s health at the psychological level, and potential social roles and cultural identity at the social level. Physiological disruptions primarily stem from cancer treatments (gonadotoxicity from chemotherapy) and reproductive capacity alterations (impaired spermatogenesis), directly triggering concerns about biological fertility loss. Psychological distress is dominated by fertility-related anxieties (genetic risks to offspring) and maladaptive cognitions (disease recurrence fears), amplified by unmet information needs. Social role conflicts arise from strained partner relationships (communication barriers) and sociodemographic pressures (unmarried/childless status), compounded by inadequate peer support.

### 6.2 Analysis influencing factors of reproductive concerns in reproductive-aged male patients with cancer

The systematic review indicates that younger, unmarried, and childless male patients with cancer exhibit higher levels of reproductive concerns (19, 20). Healthcare providers should offer accurate information about the impact of cancer treatment on fertility and potential fertility outcomes before initiating cancer treatment (32). The primary location and pathological staging of cancer influence the treatment method and prognosis; chemotherapy and radiotherapy, particularly those detrimental to fertility, indirectly raise reproductive concerns among reproductive-age patients (32). Additionally, alterations in sex hormone levels induced by chemotherapy and radiotherapy contribute to the elevated levels of reproductive concerns among male patients with cancer (46). Healthcare providers should acknowledge these reproductive concerns and assist patients in planning for fertility before and after cancer treatment. Educating patients with higher educational backgrounds on sperm cryopreservation, the gold standard for maintaining male fertility post-cancer survival, and promoting this knowledge to patients with lower educational backgrounds can most effectively address individual requirements (39).

Patients who desire children may experience reproductive concerns and confusion about their social roles due to their cancer diagnosis (21). Therefore, fertility counseling and assisted reproductive technology are essential for this population. The predominant fertility-related concern among men is the health of their present or future children (22–24). Clinicians should inform patients regarding disease factors and genetic traits to alleviate anxiety. For genetically predisposed diseases, early screening and prevention are essential (31). Patients should be thoroughly informed about the hereditary probability of diseases and the importance of health check-ups for their children (47).

Male patients with cancer who have limited peer support often experience high reproductive concerns and feel isolated and helpless. Healthcare providers must acknowledge the significance of peer support, enhance public education to eradicate cancer-related stigma, and facilitate society in comprehending male patients with cancer, consequently alleviating psychological distress and encouraging patients to pursue peer support. Furthermore, the quality of partner relationships significantly affects fertility anxiety levels (28). Patients should be informed that a positive partner relationship can alleviate negative psychological impacts caused by impaired fertility. Encouraging patients to communicate actively with their partners and confront fertility pressure together is crucial. Additionally, seeking assistance from society, where adequate social support can reduce stress, acquire information and resources, and improve coping skills, is advantageous (48).

The current status of fertility counseling for reproductive-aged male patients with cancer is not optimistic (27). Lack of multidisciplinary communication restricts reproductive medicine specialists from providing fertility counseling to patients with cancer in oncology wards, significantly impacting the quality and effectiveness of such counseling (49). Preserving male fertility is generally simpler and non-invasive compared to female. Before cancer treatment, comprehensive elucidations of the fertility preservation procedure and benefits should be conveyed. As many patients and healthcare providers lack knowledge about fertility preservation, it is imperative to strengthen the promotion of fertility preservation knowledge among healthcare providers and patients, enabling more reproductive-aged male patients with cancer to benefit (47).

For patients with cancer exhibiting elevated disease perception and anxiety regarding disease progression, prompt psychological counseling is crucial to improve their self-efficacy and perceive more support (50). Healthcare providers should encourage to self-express appropriately and effectively, especially with other patients and family members (19). Reports from the United States indicate that reproductive-age male patients also require positive psychological therapy to reduce the negative impact of reproductive concerns and enhance overall health outcomes. However, empirical studies regarding the effect of positive psychological therapy on reproductive concerns among reproductive-aged male patients with cancer are currently insufficient (51).

### 6.3 Current interventions for reproductive concerns in male cancer survivors

Current interventions for reproductive concerns in reproductive-aged male patients with cancer include fertility preservation and protection, dissemination of fertility knowledge, varied forms of fertility health information support, and psychological and social support (32).

Fertility preservation is crucial for male patients with cancer and fertility requirements. Methods of male fertility preservation encompass autologous semen cryopreservation, testicular tissue cryopreservation, and stem cell preservation (52). Autologous semen cryopreservation before the onset of chemotherapy or radiotherapy is the most prevalent and established method. Testicular tissue cryopreservation is increasingly implemented in clinical practice, and stem cell technology offers novel alternatives for azoospermic patients (32). In addition to fertility preservation, fertility protection encompasses lifestyle adjustments, supplementation with fertility-related vitamins, gonadal protection during radiotherapy, and gonadal protection using gonadotropin-releasing hormone analogs (41).

Studies have indicated that many patients lack awareness about fertility preservation methods and locations. The patients’ average score for fertility preservation knowledge was 3.5 out of 8.0, suggesting a low level of knowledge (38). Research reveals that certain oncologists hesitate to address fertility issues with patients. The knowledge level of healthcare providers regarding fertility preservation and the depth of discussions with patients also significantly affect patients’ acquisition of fertility preservation knowledge (53). Fertility health information is a fundamental necessity for reproductive-aged male patients with cancer (32). Active interventions to address this need can eliminate fertility concerns. A Swedish study integrating online lectures, exercise guidance, and expert forums to reduce fertility anxiety levels achieved positive outcomes (54). Moreover, combining online and offline methodologies can expand patient access to fertility health information (38).

Positive psychological therapy aims to alleviate negative emotions induced by fertility issues among reproductive-age patients, indirectly reducing fertility anxiety levels (50). Although positive psychological treatment has been effectively implemented for reproductive-aged female patients with favorable outcomes, empirical studies on its effects on reproductive concerns among reproductive-aged male patients with cancer are currently insufficient (51). Future research should investigate the application of positive psychological therapy in this population to offer multiple strategies to mitigate reproductive concerns among reproductive-aged male patients with cancer.

## 7 Conclusion

This systematic review demonstrated that the overall health outcomes of reproductive-aged male patients with cancer are affected by the moderately high prevalence of reproductive concerns. Gender differences in reproductive concerns frequently result in different coping characteristics among male patients. Compared to female patients, reproductive concerns in male patients are more easily overlooked, leading to physical and mental problems and a decline in the overall quality of life. Multiple factors influence reproductive concerns in reproductive-aged patients with cancer, and the lack of comprehensive intervention procedures further limits the smooth progress of clinical-related work. Most contemporary research on reproductive concerns among reproductive-aged male patients with cancer is single-center, small-sample, cross-sectional studies in single populations, with few targeted intervention studies. In the future, the following should be carried out: (1) Carry out large-scale, multi-center epidemiological studies to comprehensively assess the current situation and influencing factors of fertility concerns among patients with different types of cancer in different regions. The target population should be high-risk groups: unmarried individuals under 30 years old without children. Those who have received highly reproductive-toxic treatments (such as alkylating agent chemotherapy, pelvic radiotherapy); Key intervention time window: Diagnosis period: Conduct fertility preservation counseling. Treatment period (after each chemotherapy cycle): Psychological resilience monitoring. Survival period (1 year after treatment): Re-evaluate fertility plans; (2) Most of the existing studies are cross-sectional surveys and lack longitudinal follow-up data. In the future, the tracking research on the dynamic changes of reproductive concerns of patients at each stage from diagnosis, treatment to rehabilitation should be strengthened to explore the long-term evolution of reproductive concerns and its interrelationship with quality of life and mental health. (3) At present, there are limited intervention measures for addressing the reproductive concerns of cancer patients. In the future, high-quality intervention studies should be carried out to verify the effectiveness and feasibility of comprehensive intervention measures such as psychological support, educational guidance, and assisted reproductive counseling. (4) Medical staff, patients and caregivers should have more comprehensive communication on fertility management issues. The active cooperation of multiple disciplines is needed to promote the systematic implementation of fertility counseling and preservation techniques. (5) The future should break down the barriers between oncology, reproductive medicine and psychology: Oncology should initiate fertility risk warning; reproductive experts should be integrated into pre-treatment consultations; psychologists should provide cognitive behavioral therapy; nursing staff should lead patient-participatory education; policy levers should incorporate reproductive care into the quality control indicators of oncology.

### 7.1 Limitations

Most of the included studies in this review adopted a cross-sectional design, which--while efficient and well-suited for assessing prevalence and associations at a single time point-inevitably limits the interpretation of causal relationships. Cross-sectional studies cannot establish temporal sequencing between exposures and outcomes; thus, although associations were observed between various factors (age, marital status, fertility preservation counseling) and reproductive concerns, it remains unclear whether these variables are antecedents or consequences of such concerns. Nevertheless, the cross-sectional approach offers several strengths: it allows for rapid data collection in a cost-effective manner, facilitates the identification of at-risk subgroups, and provides foundational evidence for hypothesis generation. However, this design captures only a static snapshot and fails to reflect the evolving nature of reproductive concerns throughout the cancer trajectory--from diagnosis and treatment to survivorship and family planning. Furthermore, the reliance on self-reported measures introduces potential recall and social desirability biases, particularly given the sensitivity of fertility-related topics. The lack of standardized assessment tools across studies also complicates data synthesis and limits comparability. To advance the field, future research should adopt longitudinal or mixed-methods designs to explore the dynamic progression of reproductive concerns and better delineate causal pathways. Such approaches would enable more precise, time-sensitive interventions and contribute to the development of individualized fertility care plans for male cancer survivors of reproductive age.

Although the inclusion and exclusion criteria were strictly limited, this study still has certain limitations. (1) The sample size included in this study was relatively small, and grey literature was not included. The results may be heterogeneous. In the future, more studies related to the fertility of men with cancer during the reproductive age are needed. (2) Due to language limitations, this study was only published in Chinese and English, which limited the comprehensiveness of the research results. (3) This study was only a descriptive analysis and did not include qualitative research. A comprehensive analysis of both qualitative and quantitative research should be conducted in the future.

### 7.2 Clinical implications

The global cancer risk is rising year by year, and there is an increasing number of young cancer patients. Cancer itself and cancer treatment may have an impact on male fertility, leading to a certain degree of reproductive concerns. This study reviews the current situation and influencing factors of reproductive concerns in reproductive-aged male patients with cancer, aiming to identify high-risk patients at an early stage and explore the potential support needs of this population. The findings establish a theoretical foundation for developing comprehensive and personalized intervention measures for male cancer patients during the reproductive aged.

## Footnotes

1
https://www.crd.york.ac.uk/prospero/display_record.php?ID=CRD42024565381
