# Supplementary material for: Influencing factors of reproductive concerns in reproductive-aged male patients with cancer: a systematic review
Source: Front Public Health. 2026 May 1;14:1803648. doi: 10.3389/fpubh.2026.1803648 (PMC13176251; doi:10.3389/fpubh.2026.1803648)
Supplement: Supplementary file 1 [file Data_Sheet_1.PDF]

Influencing factors of reproductive concerns in male cancer survivors of reproductive-age: A systematic review

To enable PROSPERO to focus on COVID-19 submissions, this registration record has undergone basic automated checks for eligibility and is published exactly as submitted. PROSPERO has never provided peer review, and usual checking by the PROSPERO team does not endorse content. Therefore, automatically published records should be treated as any other PROSPERO registration. Further detail is provided here.

Citation

lu xueyu, YU yawen, XU Qiulian. Influencing factors of reproductive concerns in male cancer survivors of reproductive-age: A systematic review. PROSPERO 2024 CRD42024565381 Available from:
https://www.crd.york.ac.uk/prospero/display_record.php?ID=CRD42024565381

Review question

P:male cancer survivors of reproductive-age.

I:Psychological experiences related to reproductive concerns in young cancer patients.

C:Cancer and its treatment can cause fertility related problems in patients.

O:Degree of reproductive concerns(RCAC scale).

Searches

A comprehensive search was conducted in PubMed, the Cochrane Library, Web of Science, Embase, MEDLINE, CNKI, Wanfang Database, and VIP Database. The search period was from the inception of the databases to April 2024.

Types of study to be included

This study inclusion Observational studies (case-control studies, cohort studies, descriptive studies, etc.)

Condition or domain being studied

The research field is the mental health of male cancer patients of reproductive age, focusing on the status quo and influencing factors of fertility anxiety of male cancer patients of reproductive age, providing a theoretical basis for the intervention process of this population.

Participants/population

The population evaluated in this system was male cancer survivors of reproductive-age.

The inclusion criteria were as follows: (a)Studies on male cancer patients confirmed by pathological examination; (b)Patients aged between 18 and 50 years;

Exclusion criteria included: (a) Studies with insufficient data; (b)Non-English and non-Chinese studies; (c)Conference papers, duplicate publications, incomplete information, or studies without full-text access.

Intervention(s), exposure(s)

The phenomenon of interest in this study is the psychological experience associated with fertility anxiety in young cancer patients and cancer treatment can cause fertility related problems in patients.

The inclusion criteria were as follows: Observational studies (case-control studies, cohort studies, descriptive studies, etc.)

Comparator(s)/control

This study was a systematic review of influencing factors without intervention measures

Main outcome(s)

The main outcome index of this study was the degree of fertility anxiety.Measurement tools for fertility concerns included the Reproductive Concerns After Cancer Scale (RCAC), the Fertility Issues and Outcomes Scale (FIS), and the Reproductive Concerns Scale (RCS).

Additional outcome(s)

Secondary outcome measures were the degree of anxiety, depression, and some negative emotional situations.

Data extraction (selection and coding)

Two researchers independently screened the literature and extracted data based on the inclusion and exclusion criteria. Discrepancies were resolved through discussion or by consulting a third independent researcher. Extracted data included author, publication year, country, study population, study type, patient age, research tools, and factors related to fertility concerns.

Risk of bias (quality) assessment

The quality of each eligible article was independently assessed by two reviewers using the Strengthening the Reporting of Observational Studies in Epidemiology (STROBE) guidelines (University of Bern, 2009). This guideline includes 22 items for evaluating the quality of cross-sectional and case-control studies. Each item was scored 1 point if it met the guidelines, and 0 points if inadequately described. The total possible score was 22. Studies scoring ≥17 points were considered high-quality, those scoring 11-16 points were considered moderate-quality, and those scoring≤10 points were considered low-quality. The 13 included studies were all rated as high-quality .

Strategy for data synthesis

A comprehensive search was conducted in PubMed, the Cochrane Library, Web of Science, Embase, MEDLINE, CNKI, Wanfang Database, and VIP Database. The search period was from the inception of the databases to April 2024. Both subject terms and free terms were used. To minimize the risk of missing relevant studies, references of the included literature and related systematic reviews were tracked. The search terms included “cancer, tumor, malignancy, carcinoma, male, man, male adolescents and young adults in cancer survivorship, cancer survivors, reproductive anxiety, reproductive concerns, fertility worries, fertility anxiety, and fertility concerns”.

Analysis of subgroups or subsets

There was no subgroup analysis in this study.

Contact details for further information

lu xueyu

lxueyu61@gmail.com

Organisational affiliation of the review

Yangzhou University

Review team members and their organisational affiliations

Miss lu xueyu. Yangzhou University

Miss YU yawen. Yangzhou University

Miss XU Qiulian. Yangzhou University

Type and method of review

Systematic review

Anticipated or actual start date

01 June 2024

Anticipated completion date

31 December 2024

Funding sources/sponsors

This study was not funded.

Conflicts of interest

Language

English

Country

China

Stage of review

Review Ongoing

Subject index terms status

Subject indexing assigned by CRD

Subject index terms

MeSH headings have not been applied to this record

Date of registration in PROSPERO

14 July 2024

Date of first submission

04 July 2024

Stage of review at time of this submission

Stage	Started	Completed
Preliminary searches	Yes	Yes
Piloting of the study selection process	Yes	Yes
Formal screening of search results against eligibility criteria	Yes	No
Data extraction	No	No
Risk of bias (quality) assessment	No	No
Data analysis	No	No

The record owner confirms that the information they have supplied for this submission is accurate and complete and they understand that deliberate provision of inaccurate information or omission of data may be construed as scientific misconduct.

The record owner confirms that they will update the status of the review when it is completed and will add publication details in due course.

Versions

14 July 2024

14 July 2024
